# Association of Phthalate Exposure with Thyroid Function and Thyroid Homeostasis Parameters in Type 2 Diabetes

**DOI:** 10.1155/2021/4027380

**Published:** 2021-10-27

**Authors:** Yi Chen, Wen Zhang, JingSi Chen, Ningjian Wang, Chi Chen, Yuying Wang, Heng Wan, Bo Chen, Yingli Lu

**Affiliations:** ^1^Institute and Department of Endocrinology and Metabolism, Shanghai Ninth People's Hospital, Shanghai JiaoTong University School of Medicine, Shanghai, China; ^2^Key Laboratory of Public Health Safety of Ministry of Education, Collaborative Innovation Center of Social Risks Governance in Health, School of Public Health, Fudan University, Shanghai, China

## Abstract

**Aims:**

Phthalates, which are recognized environmental endocrine-disrupting chemicals, are associated with thyroid hormone disruption. We aimed to evaluate the relationship of phthalate metabolites with thyroid function and thyroid homeostasis parameters in type 2 diabetes and to explore whether thyroid autoimmunity status and metformin, the most common antidiabetic drug, may influence such associations.

**Methods:**

Concurrent urine and blood samples were collected from 639 participants with type 2 diabetes in the METAL (Environmental Pollutant Exposure and Metabolic Diseases in Shanghai) study. We measured urinary concentrations of thirteen phthalate metabolites along with serum levels of thyroid-stimulating hormone (TSH), total T_4_ and T_3_, free T_4_ (FT_4_) and T_3_ (FT_3_), thyroid peroxidase antibody (TPOAb), and thyroglobulin antibody (TgAb). Four parameters of thyroid homeostasis, including the sum activity of step-up deiodinases (SPINA-GD), thyroid secretory capacity (SPINA-GT), Jostel's TSH index (TSHI), and thyrotroph thyroid hormone resistance index (TTSI), were also calculated.

**Results:**

Among all participants, after full adjustment, multivariable regression analysis showed that some urine phthalate metabolites were negatively associated with TSH, TSHI, and TTSI levels and positively associated with FT_3_, T_3_, SPINA-G_D_, and SPINA-G_T_ levels. None of the urine phthalate metabolites exhibited a significant association with thyroid autoimmunity. The associations of phthalate metabolites with thyroid function and thyroid homeostasis parameters differed based on thyroid autoantibody and metformin treatment status.

**Conclusions:**

Urinary phthalate metabolites may be associated with thyroid function and thyroid homeostasis parameters among participants with type 2 diabetes. Furthermore, our present study suggested that thyroid autoantibody status and metformin treatment status are potential mediators of such associations.

## 1. Introduction

Phthalates, a group of similar diesters of phthalic acid, are ubiquitous in different environmental exposure media around the world. Human exposure to phthalates mainly occurs through food due to their use during food production, processing, and packaging [1, 2]. Other important phthalate exposure routes involve inhalation of contaminated air, application of cosmetics, medical devices, and personal care products [3, 4]. China is currently facing relatively higher environmental and food contamination problems, especially in the Yangtze River Delta areas (including Shanghai) [1].

Phthalates are recognized environmental endocrine-disrupting chemicals, and one of their well-known target organs is the thyroid. Thyroid hormones play vital roles in human growth, brain development, energy metabolism, and reproductive systems [5, 6] and participate in regulating various processes of lipid and glucose metabolism [7, 8]. Recently, studies have demonstrated that thyroid-stimulating hormone (TSH) levels within the reference range were also associated with neurocognitive problems [9], obesity, lipid profile, insulin resistance, and other risk factors for cardiovascular diseases [10, 11]. The literature has reported associations between different phthalate metabolites and thyroid hormone levels in both children and adults with inconsistent results [12–14]. Animal studies have indicated that phthalates alter thyroid signaling through a number of potential mechanisms, including interference with the TSH receptor, binding to transport proteins, interfering with the hypothalamic-pituitary-thyroid axis, and changing sodium-iodide symporter-mediated iodide uptake, iodothyronine deiodinases, or hepatic enzymes [12, 15–19].

To the best of our knowledge, evidence of thyroid disruption as a result of phthalate exposure among participants with type 2 diabetes is also very limited. Furthermore, metformin is the most commonly used antidiabetic drug in the world. Previous researchers have reported that metformin acts as an endocrine-disrupting compound (EDC) [20–22]. However, related studies in the human population have rarely been reported. Therefore, in the current study, we aimed to evaluate the relationship of phthalate metabolites with thyroid function variables and thyroid homeostasis parameters and to explore whether thyroid autoimmunity status and metformin treatment may influence such associations.

## 2. Methods

### 2.1. Study Design and Participants

Our study population was derived from a cross-sectional study performed in 2018, the METAL study [23] (Environmental Pollutant Exposure and Metabolic Diseases in Shanghai, http://www.chictr.org.cn/, ChiCTR1800017573), which enrolled diabetic patients from seven communities in Huangpu and Pudong District in Shanghai, China. The METAL study randomly selected half of diabetic patients from the registration platform of each community health care centre. Participants were Chinese diabetic citizens ≥ 18 years old who had lived in their current area for ≥6 months. Among the seven communities, one community patient (*n* = 698) was invited to produce fasting spot urine samples for the measurement of phthalate metabolites. Participants who were missing thyroid function parameters (*n* = 2), had a history of thyroid diseases (including thyroid surgery history and hyperthyroidism, hypothyroidism, and radioactive iodine treatment history) (*n* = 40), used amiodarone (*n* = 3), or are missing urine creatinine (Cr) values (*n* = 14) were excluded from this study. Finally, 639 subjects were included in the final analysis. All participants provided written informed consent before data collection. The study protocol was approved by the Ethics Committee of Shanghai Ninth People's Hospital, Shanghai JiaoTong University School of Medicine. All procedures were performed in accordance with the ethical standards of the responsible committee on human experimentation (institutional and national) and with the Helsinki Declaration of 1975 and revised in 2008.

### 2.2. Data Collection

At every step of this study, all data collection was performed by the same staff group in the SPECT-China study [24] from the Department of Endocrinology and Metabolism in Shanghai Ninth People's Hospital, Shanghai JiaoTong University School of Medicine. A standard questionnaire was administered by trained staff to obtain information on demographic characteristics, lifestyle habits (e.g., smoking status), medical history, medication prescribed for type 2 diabetes, duration of diabetes, and thyroid diseases. Weight, height, and blood pressure were measured according to a standard protocol.

### 2.3. Laboratorial Measurements

#### 2.3.1. Serum Samples

Serum samples for laboratory assays were obtained by venipuncture after an 8-hour fast. Blood samples were stored at 2-8°C when collected and shipped to one central laboratory, which was certified by the College of American Pathologists (CAP), within 2-4 hours of collection. On the day of blood sample collection, as soon as the blood samples arrived in the laboratory, serum separation and sample testing began. Serum TSH, free triiodothyronine (FT_3_), free thyroxine (FT_4_), total triiodothyronine (T_3_), total thyroxine (T_4_), thyroid peroxidase antibody (TPOAb), and thyroglobulin antibody (TgAb) were measured by electrochemiluminescence (Roche, E601, Germany). The normal reference ranges for TSH, FT_3_, FT_4_, T_3_, T_4_, TPOAb, and TgAb were 0.27~4.20 mIU/L, 3.10~6.80 nmol/L, 12.00~22.00 nmol/L, 1.30-3.10 nmol/L, 66.00-181.00 nmol/L, 0~34.00 IU/L, and 0~115.00 IU/L, respectively. Two structure parameters of thyroid homeostasis, SPINA-G_D,_ and SPINA-G_T_, as well as two pituitary thyrotropic function indices, TSHI and TTSI, were calculated according to previous studies [25].

#### 2.3.2. Urine Sample Collection and Measurement of Urinary Metabolites of Phthalates

Spot urine was obtained from each participant in the same morning. Using the morning fasting spot urine samples, urine iodine concentration (UIC) was determined with an inductively coupled plasma-mass spectrometry (ICP-MS) (Agilent Technologies Inc., Agilent 7700X, USA). The urine Cr was measured with a Beckman Coulter AU 680 (Brea, USA) using a turbidimetric immunoassay and an enzymatic method. In addition, thirteen urinary metabolites of phthalates were determined by using the method of ultraperformance liquid chromatography tandem mass spectrometry (UPLC-MS/MS), which included monomethyl phthalate (MMP), monoethylphthalate (MEP), monoisobutylphthalate (MiBP), mono-n-butylphthalate (MnBP), mono-benzylphthalate (MBzP), mono-2-ethyl-5-carboxypentylphthalate (MCPP), mono-isononyl phthalate (MiNP), mono-cyclohexyl phthalate (MCHP), mono-2-ethylhexylphthalate (MEHP), mono-2-ethyl-5-carboxypentylphthalate (MECPP), mono-2-ethyl-5-hydroxyhexylphthalate (MEHHP), mono-2-ethyl-5-oxohexylphthalate (MEOHP), and mono-2-carboxy-hexyl-phthalate (MCMHP). Among them, MMP, MEP, MiBP, MnBP, MBzP, MiNP, and MCHP are the metabolites of dimethyl phthalate (DMP), diethyl phthalate (DEP), di-iso-butyl phthalate (DiBP), di-n-butyl phthalate (DnBP), benzyle butyl phthalate (BBP), di-isononyl phthalate (DiNP), and di-cyclohexyl phthalate (DCHP), respectively. MCPP is a metabolite of three phthalates (DnBP, Di-n-ocytl phthalate (DnOP), and DiNP). MEHP, MEOHP, MEHHP, MECPP, and MCMHP are the metabolites of di(2-ethylhexyl)phthalate (DEHP). In brief, the following methodology was employed: The urine sample (1 mL) was incubated with *β*-glucuronidase (E. coli K 12; Roche, Mannheim, Germany) at 37°C for 120 min. The sample was subsequently acidified with 1 mL of aqueous 2% (*v*/*v*) acetic acid and mixed with 100 *μ*L of internal standard (100 *μ*g/L). The mixture was loaded onto a PLS column (Dikma, China; 60 mg/3 mL) previously activated with 2 mL methanol and 2 mL aqueous 0.5% (*v*/*v*) acetic acid. After sample loading, the column was washed with 2 mL of aqueous 0.5% (*v*/*v*) acetic acid. Then, 1 mL of methanol was added to elute metabolites. Finally, the eluate was passed through a 0.2 *μ*m filter and analysed (2 *μ*L) using a UPLC–MS/MS system integrated by a Waters ACQUITY UPLC H-Class (Waters, USA) coupled with an ABSCIEX QTRAP 6500 (AB Sciex Technologies, Framingham, MA, USA). The analytical column was a Waters ACQUITY UPLC BEH C18 Column (1.7 *μ*m, 2.1 × 50 mm, Waters, USA).

An internal standard method was used for quality control of laboratory procedures. For every 20 samples, a procedural blank and two matrix-spiked samples at two different spiking concentrations (5 and 15 ng/mL) were processed. The average recoveries and relative standard deviations (RSDs) of target metabolites ranged from 86.5% to 123.1% and from 0.5% to 12.5% at 5 *μ*g/L and from 75.3% to 98.4% and from 0.7% to 8.8% at 15 *μ*g/L, respectively. Sample concentrations of these metabolites were determined after subtraction of blank values. The limits of detection (LODs) were calculated at a signal-to-noise (S/N) of 3 with concentrations of 0.100, 0.080, 0.006, 0.006, 0.060, 0.006, 0.010, 0.020, 0.020, 0.040, 0.100, 0.010, and 0.004 *μ*g/L for MMP, MEP, MnBP, MiBP, MBzP, MEHP, MEOHP, MEHHP, MECPP, MCMHP, MCPP, MiNP, and MCHP, respectively [26]. The results below the LOD were substituted with a value of the LOD divided by 2.

### 2.4. Statistical Analysis

We performed survey analyses with IBM SPSS Statistics, Version 22 (IBM Corporation, Armonk, NY, USA). All analyses were two-sided. A *P* value < 0.05 was considered significant. The Kolmogorov-Smirnov test and P-P plots were used to determine whether the data were normally distributed. Normally distributed variables are expressed as the mean ± standard deviation, whereas variables with a nonnormal distribution are reported as the median (interquartile range). Categorical variables are presented as percentages. Normally distributed continuous variables were compared using Student's *t*-test. The Mann–Whitney *U* test was used for nonnormally distributed continuous variables. The Pearson chi-squared test was used for dichotomous variables.

Linear and logistic regression analyses were performed to assess the associations of urinary phthalates with thyroid function, thyroid autoimmunity, and thyroid homeostasis parameters. In the multiple regression analysis, covariates of age, sex (male or female), BMI, current smoking status, and UIC were included. Before analysis, urinary concentrations of phthalate metabolites and UIC were corrected by urine Cr and ln-transformed to approximate a normal distribution. As one chemical was considered at a time in the regression analysis, this model was termed a single-chemical model. The results were expressed as *B* values and 95% confidence intervals (CIs) and odds ratios (ORs) and 95% CIs. To evaluate the effect of exposure to multiple chemicals, multiple-chemical models were analysed. Factor analysis of dimension reduction was performed to reduce the number of variables. Subsequently, four components were chosen with the MINEIGEN criterion (eigenvalues are greater than one). Factor loadings greater than 0.5 were considered highly loaded. These four factors were then included in the further linear regression analysis after varimax rotation along with all the other covariates.

## 3. Results

### 3.1. Participant's Clinical Characteristics

Table 1 shows the clinical characteristics and serum thyroid measures of the study population. A total of 639 diabetic participants (321 men and 318 women) were enrolled in this study. The mean age was 68.51 ± 8.09 years, and the mean BMI was 25.00 ± 3.54 kg/m^2^. Among all the participants, TPOAb and TgAb were positive in 6.1% and 4.2% of the study population, respectively, and 8.5% of the study population had TPOAb and/or TgAb positivity. Most of the subjects had normal TSH levels; however, 15.3% and 0.5% of the participants had higher and lower TSH levels, respectively, than the reference range.

### 3.2. Urinary Concentrations of Phthalate Metabolites

The urinary concentrations of the determined metabolites of the aforementioned phthalates are also shown in Table 1. Among non-DEHP metabolites (MMP, MEP, MiBP, MnBP, MBzP, MCPP, MiNP, and MCHP), MnBP had the highest concentration (142.70 *μ*g/g) followed by MiBP (86.04 *μ*g/g). MCHP had the lowest concentration (0.05 *μ*g/g) followed by MBzP (0.92 *μ*g/g). For DEHP metabolites, MECPP exhibited the highest concentration (32.29 *μ*g/g) followed by MEHHP (24.41 *μ*g/g) and MEOHP (12.57 *μ*g/g). Most concentrations of urinary phthalate metabolites were higher in women compared with men, except for MMP, MCHP, and MEHP.

### 3.3. Associations between Urinary Phthalates and Thyroid Parameters: Single-Chemical Model

After adjusting for age, sex (male or female), BMI, current smoking status, and urine iodine-to-creatinine ratio (UI/Cr), linear and logistic regression analyses were performed to assess the associations of urinary phthalates with thyroid function, thyroid autoimmunity, and thyroid homeostasis parameters using single-chemical models. Multivariable regression analysis showed negative associations between urine phthalate metabolites (MiNP, MEHHP, and MEOHP) and TSH levels (MiNP: *B* = −0.057, 95% CI -0.112, -0.003, *P* = 0.040; MEHHP: *B* = −0.063, 95% CI -0.118, -0.008, *P* = 0.025; and MEOHP: *B* = −0.045, 95% CI -0.089, -0.001, *P* = 0.046). MCMHP showed positive associations with FT_3_ and T_3_ (FT_3_: *B* = 0.050, 95% CI 0.009, 0.092, *P* = 0.017; T_3_: *B* = 0.026, 95% CI 0.006, 0.047, *P* = 0.013). MECPP also showed a positive association with T_3_ (*B* = 0.024, 95% CI 0.002, 0.047, *P* = 0.036) (Table 2). In terms of thyroid autoimmunity, none of the urine phthalate metabolites showed a significant association with an increased risk of TPOAb, TgAb, and TPOAb prevalence and/or TgAb positivity (all *P* > 0.05). However, when classified by the presence of thyroid antibodies (TPOAb and/or TgAb positivity), the associations of phthalate metabolites with thyroid function differed (Table 3). Table 2 shows the relationship between urinary phthalates and four thyroid homeostasis parameters. After full adjustment, MECPP, MEHHP, and MCMHP showed positive associations with SPINA-G_D_. MiNP, MEHP, and MEHHP were positively associated with SPINA-G_T_. MiNP, MEHHP, MEOHP, and MCMHP exhibited negative associations with TSHI and TTSI (all *P* < 0.05). The associations of phthalate metabolites observed with these thyroid homeostasis parameters also differed based on thyroid autoantibody status (Table 3).

### 3.4. Associations between Urinary Phthalates and Thyroid Parameters: Multiple-Chemical Model

Factor analysis was performed to identify major factors due to exposure to multiple chemicals simultaneously. Factor analysis showed that four factors explained 37.58%, 20.72%, 8.28%, and 7.79% of the variance, respectively. Factor 1 was highly loaded with MECPP, MEHHP, MEOHP, MCMHP, and MiNP. Factor 2 was highly loaded with MiBP, MnBP, and MEHP. Factor 3 was highly loaded with MCPP and MCHP, and Factor 4 was highly loaded with MBzP. Then, multifactor regression analysis was performed after adjusting for age, sex, BMI, smoking status, UI/Cr, and Factors 1, 2, 3, and 4. Factor 1 was negatively associated with TSH (*B* = −0.056, 95% CI -0.107, -0.004, *P* = 0.034), TSHI (*B* = −0.062, 95% CI -0.114, -0.011, *P* = 0.017), and TTSI (*B* = −0.060, 95% CI -0.110, -0.010, *P* = 0.019) but positively associated with SPINA-G_T_ (*B* = 0.043, 95% CI 0.007, 0.079, *P* = 0.018). No other significant association was found (Table 4).

### 3.5. Associations between Urinary Phthalates and Thyroid Parameters Based on Metformin Treatment Status

Among all the participants, 272 participants (42.6%) were currently using metformin treatment. We then evaluated the associations between urinary phthalates and thyroid parameters among the participants with or without metformin treatment. We found that the associations between urinary phthalates and serum thyroid parameters, such as FT_4_, T_4_, T_3_, and TTSI, differed based on metformin treatment status. Urinary phthalates showed significant associations with TSH and SPINA-G_T_ only among those without metformin treatment, whereas significant associations with FT_3_, SPINA-G_D_, and TSHI were only among those with metformin treatment (Table 5).

## 4. Discussion

In the current study, we evaluated the relationship of thirteen phthalate metabolites with thyroid function variables (TSH, FT_3_, FT_4_, T_3_, and T_4_) and thyroid homeostasis parameters (SPINA-G_D_, SPINA-G_T_, TSHI, and TTSI) among participants with type 2 diabetes in the METAL study (2018) in Shanghai, China. We found that some of the urine phthalate metabolites were negatively associated with TSH, TSHI, and TTSI levels and positively associated with FT_3_, T_3_, SPINA-G_D_, and SPINA-G_T_ levels. Additionally, our present study suggested that thyroid autoimmunity status and metformin treatment status might be potential mediators of the association between urinary phthalate metabolites and thyroid function.

Previous studies have shown that some of the phthalate metabolites were associated with age and anthropometric measurements, including height, weight, and BMI [12], so it seems that age and BMI may affect phthalate metabolism [13]. In addition, considering the effect of iodine levels [27] and smoking status [28] on thyroid function, all analyses in this study were performed with adjustment for age, BMI, smoking status, and urine iodine to creatinine ratio. It was reported that urinary concentrations of phthalate metabolites could be determined using the urinary Cr correction approach as well as the urine volume approach [29, 30], and these two methods can accurately reflect phthalate metabolism and excretion [13]. Therefore, all phthalate metabolites are divided by urinary Cr for correction.

In the present study, we measured thirteen urinary metabolites of phthalates, including eight non-DEHP metabolites (MMP, MEP, MiBP, MnBP, MBzP, MCPP, MiNP, and MCHP) and five DEHP metabolites (MEHP, MECPP, MEHHP, MEOHP, and MCMHP). Although MEHP, the primary metabolite of DEHP, was widely used as a recognized biomarker to predict exposure to DEHP, the major metabolites of DEHP in this study were MECPP followed by MEHHP, MEOHP, and MCMHP, which was consistent with other studies [1, 31–33]. Thus, in recent studies, oxidative secondary metabolites (mainly MECPP, MEOHP, and MEHHP) along with primary metabolites (MEHP) have also been used to predict DEHP exposure [33–35].

Several studies have reported inconsistent associations between urine phthalate metabolites and thyroid hormone levels [14, 36, 37]. In NHANES 2007–2008, Meeker and Ferguson observed significant inverse relationships between MEHHP, MEOHP, or MECPP and T_4_, FT_4_, and T_3_ and positive relationships with TSH [14]. In the Korean National Environmental Health Survey (KoNEHS) 2015–2017, phthalate metabolites were negatively associated with T_4_ and FT_3_ and positively associated with T_3_ [36]. In the current study, we found that phthalate metabolites were negatively associated with TSH and positively associated with FT_3_ and T_3_. Factor analysis was then performed to identify major factors. Major factors that were identified from the factor analysis demonstrated that target chemicals could be classified based on the shared characteristics of exposure. The results of the multiple-chemical models, which included major factors identified from the factor analysis, showed that phthalate metabolites, i.e., Factor 1, which was highly loaded with MECPP, MEHHP, MEOHP, MCMHP, and MiNP, were negatively associated with TSH levels.

A recent study reported that calculated peripheral deiodinase (DIO) activity was positively associated with phthalate metabolites [36]. In the current study, we explored the relationship of phthalate metabolites with thyroid homeostasis parameters, including two structural parameters of thyroid homeostasis (SPINA-G_D_ and SPINA-G_T_) and two pituitary thyrotrophic function indices (TSHI and TTSI). By extending the classic concept of separate measurements of thyroid hormone parameters, these markers add new qualitative and quantitative dimensions to the evaluation of thyroid homeostasis and deliver important insights into the physiology of pituitary-thyroid feedback control [25]. We found that urine phthalate metabolite levels were positively associated with SPINA-G_D_ and SPINA-G_T_ and negatively associated with TSHI and TTSI. Thus, we suggest that phthalate exposure may be associated with enhancing the maximum stimulated activity of step-up deiodination and the maximum secretion rate of the thyroid gland under stimulated conditions and reducing the thyrotrophic function of the pituitary gland [25, 38].

Autoimmune thyroid diseases (AITDs) are characterized by progressive destruction of thyroid tissue that may lead to thyroid disorders (i.e., subclinical hypothyroidism and overt hypothyroidism) [39, 40]. High levels of either TPOAb or TgAb serve as a clinical marker for the detection of AITD [39, 41]. In previous animal studies, DBP treatment of rats increased the production of Tg antibody and chronic lymphocytic thyroiditis induced by Tg [42]. However, studies about the association of urinary phthalate metabolites and thyroid antibodies have been reported in the human population, especially in diabetes participants. Data from the National Health and Nutrition Examination Survey (NHANES) 2007-2008 suggested that inverse relationships existed between urinary DEHP metabolites and TgAb [14], whereas another study in the Korean National Environmental Health Survey (KoNEHS) from 2015 to 2017 reported that no direct associations were observed between chemical exposure and thyroid autoantibody status [36]. In the current study, among these type 2 diabetes participants, although none of the urine phthalate metabolites showed a significant association with TPOAb, TgAb, and TPOAb and/or TgAb positivity, different associations between the presence of thyroid antibodies and TSH, FT_3_, FT_4_, T_4_, SPINA-G_D_, SPINA-G_T_, TSHI, and TTSI were found. Our observations suggested potential mediating effects of thyroid autoantibody status on the relationship of phthalate metabolites with thyroid function and thyroid homeostasis. However, given that cross-sectional studies are unable to establish a causal relationship along with the limitations of a small number of subjects who are thyroid antibody-positive, further studies are needed.

Among all the participants, 272 participants (42.6%) were currently using metformin treatment. As a first-line treatment for type 2 diabetes, metformin was reported to have some effect on the hypothalamic-pituitary-thyroid axis [43] and is also associated with a decrease in the levels of TSH possibly by enhancing the effects of thyroid hormones in the pituitary and activating adenosine monophosphate-activated protein kinase (AMPK) [44]. Associations between phthalate exposure and metformin treatment status have rarely been assessed in the human population. In the current study, we were surprised to find that the associations between urinary phthalates and serum thyroid parameters (i.e., FT_4_, T_4_, T_3_, and TTSI) differed based on metformin treatment status. Most significant associations between phthalate metabolites, especially DEHP metabolites (i.e., MECPP, MEHHP, MEOHP, and MCMHP), and thyroid-related parameters were found among participants undergoing metformin treatment. A possibility to be considered includes mitochondrial toxicity exerted by phthalate metabolites [45–47]. Impaired function of the respiratory chain leads to the accumulation of reduced forms of redox coenzymes, including NADH and NADPH [48]. This process occurs primarily within the mitochondria, but via FAD, malate and glycerol phosphate shuttles cause the ratio of reduced to oxidized coenzymes to increase in the cytosol under these conditions. Given that NADPH is an indirect cosubstrate for deiodinases (involving glutathione and other substrates), the accumulation of reduced coenzymes will result in step-up hyperdeiodination after exposure to phthalates. Therefore, this finding provides a plausible explanation for why phthalate toxicity leads to increased deiodinase activity [48]. This notion also provides an explanation of the reduced set point of thyroid homeostasis due to phthalate exposure given that the upregulation of central deiodinases leads to a suppression of hypothalamic TRH release and subsequently TSH concentration [49]. In recent years, many researchers have given attention to metformin as an endocrine disruptor and its possible mechanism. Lee et al. evaluated metformin aquatic toxicity by exposing Oryzias latipes to metformin and demonstrated that metformin leads to oxidative stress and endocrine disruption in O. latipes [20]. Niemuth and Klaper measured the expression of numerous endocrine-related genes in male fathead minnows exposed to metformin at a low dose similar to that found in wastewater effluent and provided additional evidence of the endocrine-disrupting impact of metformin [22]. Our observations suggested a potential mediating effect of metformin treatment status, and the mechanism of metformin-induced endocrine disruption needs to be further explored in the human population.

In the current study, we used ultraperformance liquid chromatography tandem mass spectrometry-based measurement of urinary phthalate metabolites to generate more accurate and precise data. This novel study may be the first to evaluate the relationship of phthalate metabolites with thyroid function and thyroid homeostasis parameters in type 2 diabetes and to investigate the possible mediating effect of thyroid autoimmunity and metformin treatment status on this relationship, which will provide a new understanding of phthalate exposure as an endocrine disruptor. However, some limitations must be taken into account. First, given the cross-sectional study design, we were unable to make any causal conclusions regarding the association of phthalate exposure and thyroid parameters. Second, only one single time point of urine and blood sample per subject was analysed, which may result in measurement error and bias the results of phthalate and thyroid function. Finally, humans are constantly and simultaneously exposed to many environmental chemicals. Thus, due to their omnipresence in the environment, a mixture of EDCs, such as DEHP, DBP, and bisphenol A (BPA), has the capability to induce more pronounced effects than those provoked by single substances. Possible additive effects, including synergistic and antagonistic effects, were reported both in vivo and in vitro [50–53]. However, this study did not cover a variety of EDCs, and the effects of the mixture need to be clarified by future studies.

## 5. Conclusion

Among participants with type 2 diabetes, a significant association suggesting that thyroid function and homeostasis status disrupt the effects of phthalate exposure was observed. We also found suggestive evidence that thyroid autoantibody status and metformin treatment status might modulate the associations between some phthalate metabolites and thyroid function and homeostasis status.

## Figures and Tables

**Table 1:** Characteristics of the study samples.

Characteristic	Total	Men	Women	*P*
*n*	639	321	318	—
Age (yr)	68.51 ± 8.09	68.78 ± 8.52	68.25 ± 7.63	0.405
BMI (kg/m^2^)	25.00 ± 3.54	24.76 ± 3.20	25.24 ± 3.84	0.094
Smokers (%)	20.6	39.8	1.3	<0.001
HbA1c (%)	7.30 (1.70)	7.30 (1.60)	7.20 (1.70)	0.167
*Thyroid parameters*				
TSH (mIU/L)	2.42 (1.89)	2.29 (1.66)	2.69 (2.06)	0.002
FT_3_ (pmol/L)	4.56 ± 0.56	4.65 ± 0.58	4.67 ± 0.53	<0.001
FT_4_ (pmol/L)	16.29 ± 2.13	16.44 ± 2.07	16.13 ± 2.18	0.068
T_3_ (nmol/L)	1.61 ± 0.28	1.61 ± 0.29	1.62 ± 0.26	0.579
T_4_ (nmol/L)	97.43 ± 17.36	95.20 ± 16.91	99.69 ± 17.55	0.001
TPOAb (+) (%)	6.1	4.4	7.9	0.065
TgAb (+) (%)	4.2	0.6	7.9	<0.001
TPOAb and/or TgAb (+) (%)	8.5	4.4	12.6	<0.001
*Thyroid homeostasis parameters*				
SPINA-G_D_ (nmol/s)	15.45 ± 3.06	15.24 ± 3.06	15.67 ± 3.04	0.077
SPINA-G_T_ (pmol/s)	2.24 (1.12)	2.29 (1.13)	2.21 (1.11)	0.556
TSHI	3.06 ± 0.59	3.02 ± 0.58	3.10 ± 0.61	0.092
TTSI	181.21 (128.58)	175.01 (119.47)	195.95 (140.20)	0.009
*Non-DEHP metabolites*				
MMP (*μ*g/g)	7.17 (7.93)	7.52 (8.00)	6.99 (7.82)	0.342
MEP (*μ*g/g)	24.00 (45.86)	21.55 (35.85)	27.04 (59.34)	0.001
MiBP (*μ*g/g)	86.04 (85.49)	79.00 (80.36)	93.39 (91.23)	0.002
MnBP (*μ*g/g)	142.70 (173.46)	129.61 (148.31)	165.12 (200.56)	0.002
MBzP (*μ*g/g)	0.92 (1.28)	0.83 (1.33)	1.03 (1.24)	0.019
MCPP (*μ*g/g)	3.94 (8.42)	3.47 (8.65)	4.44 (8.54)	0.005
MiNP (*μ*g/g)	13.33 (14.20)	12.07 (12.18)	14.99 (17.09)	0.005
MCHP (*μ*g/g)	0.05 (0.32)	0.06 (0.31)	0.04 (0.32)	0.946
*DEHP metabolites*				
MEHP (*μ*g/g)	4.02 (8.37)	4.66 (8.28)	3.54 (8.51)	0.236
MECPP (*μ*g/g)	32.29 (34.83)	29.51 (30.10)	34.40 (39.73)	0.003
MEHHP (*μ*g/g)	24.41 (28.01)	22.33 (24.69)	27.27 (34.25)	<0.001
MEOHP (*μ*g/g)	12.57 (14.07)	11.15 (11.75)	14.00 (16.92)	0.01
MCMHP (*μ*g/g)	9.65 (11.70)	8.67 (11.02)	11.34 (12.68)	<0.001

Data are summarized as the median (interquartile range) or the mean ± standard deviation for continuous variables or a proportion for categorical variables. Normally distributed continuous variables are compared using Student's *t*-test. The Mann–Whitney *U* test is used for nonnormally distributed continuous variables. The Pearson chi-squared test is used for dichotomous variables.

**(a):** 

	TSH		FT_3_		FT_4_		T_3_		T_4_	
	*B* (95% CI)	*P*	*B* (95% CI)	*P*	*B* (95% CI)	*P*	*B* (95% CI)	*P*	*B* (95% CI)	*P*
MMP	-0.032 (-0.075, 0.012)	0.154	0.020 (-0.018, 0.059)	0.305	0.016 (-0.139, 0.171)	0.842	0.014 (-0.006, 0.033)	0.167	-0.030 (-1.272, 1.211)	0.962
MEP	-0.006 (-0.032, 0.021)	0.676	-0.008 (-0.031, 0.016)	0.524	0.012 (-0.082, 0.105)	0.803	-0.006 (-0.018, 0.006)	0.327	-0.337 (-1.085, 0.410)	0.476
MiBP	-0.015 (-0.081, 0.051)	0.654	0.043 (-0.016, 0.101)	0.150	-0.013 (-0.248, 0.221)	0.910	0.010 (-0.019, 0.039)	0.508	0.005 (-1.869, 1.878)	0.996
MnBP	-0.002 (-0.061, 0.056)	0.935	0.041 (-0.011, 0.093)	0.118	-0.147 (-0.355, 0.061)	0.165	0.016 (-0.010, 0.042)	0.221	-0.176 (-1.843, 1.491)	0.836
MBzP	0.014 (-0.026, 0.054)	0.502	0.010 (-0.026, 0.045)	0.596	0.001 (-0.141, 0.144)	0.985	0.012 (-0.006, 0.030)	0.179	1.028 (-1.107, 2.163)	0.076
MCPP	0.007 (-0.017, 0.030)	0.579	-0.010 (-0.031, 0.011)	0.364	-0.004 (-0.088, 0.080)	0.927	-0.009 (-0.020, 0.001)	0.087	-0.299 (-0.970, 0.372)	0.382
MiNP	-0.057 (-0.112, -0.003)	0.040	0.023 (-0.026, 0.071)	0.355	-0.051 (-0.245, 0.143)	0.604	0.017 (-0.007, 0.041)	0.172	0.396 (-1.158, 1.950)	0.617
MCHP	-0.003 (-0.024, 0.018)	0.780	0.007 (-0.012, 0.026)	0.451	-0.006 (-0.081, 0.069)	0.873	0.006 (-0.004, 0.015)	0.246	0.448 (-0.152, 1.048)	0.143
MEHP	-0.021 (-0.045, 0.002)	0.077	0.005 (-0.016, 0.026)	0.635	0.061 (-0.023, 0.145)	0.156	0.005 (-0.005, 0.016)	0.318	0.545 (-0.126, 1.215)	0.111
MECPP	-0.034 (-0.085, 0.018)	0.201	0.039 (-0.006, 0.085)	0.090	-0.112 (-0.295, 0.071)	0.230	0.024 (0.002, 0.047)	0.036	0.649 (-0.816, 2.113)	0.385
MEHHP	-0.063 (-0.118, -0.008)	0.025	0.035, -0.014, 0.084	0.157	-0.110 (-0.304, 0.085)	0.268	0.017 (-0.008, 0.041)	0.175	0.138 (-1.419, 1.696)	0.862
MEOHP	-0.045 (-0.089, -0.001)	0.046	0.001 (-0.038, 0.040)	0.954	-0.127 (-0.282, 0.029)	0.110	0.008 (-0.011, 0.028)	0.403	-0.008 (-1.257, 1.240)	0.989
MCMHP	-0.045 (-0.091, 0.002)	0.062	0.050 (0.009, 0.092)	0.017	-0.096 (-0.263, 0.070)	0.257	0.026 (0.006, 0.047)	0.013	0.166 (-1.166, 1.499)	0.807

**(b):** 

	SPINA-G_D_		SPINA-G_T_		TSHI		TTSI	
	*B* (95% CI)	*P*	*B* (95% CI)	*P*	*B* (95% CI)	*P*	*B* (95% CI)	*P*
MMP	0.141 (-0.081, 0.362)	0.212	0.020 (-0.011, 0.050)	0.205	-0.030 (-0.073, 0.014)	0.181	-0.032 (-0.074, 0.011)	0.143
MEP	-0.035 (-0.169, 0.098)	0.604	0.004 (-0.014, 0.022)	0.666	-0.004 (-0.030, 0.022)	0.764	-0.006 (-0.032, 0.020)	0.643
MiBP	0.160 (-0.173, 0.494)	0.346	0.011 (-0.035, 0.057)	0.628	-0.017 (-0.083, 0.049)	0.614	-0.018 (-0.083, 0.046)	0.579
MnBP	0.320 (0.024, 0.616)	0.034	0.001 (-0.040, 0.042)	0.975	-0.022 (-0.081, 0.036)	0.456	-0.013 (-0.070, 0.044)	0.660
MBzP	0.132 (-0.071, 0.334)	0.203	0.003 (-0.025, 0.031)	0.833	0.014 (-0.026, 0.054)	0.493	0.014 (-0.025, 0.053)	0.485
MCPP	-0.072 (-0.192, 0.047)	0.237	-0.006 (-0.022, 0.011)	0.479	0.006 (-0.017, 0.030)	0.607	0.006 (-0.017, 0.029)	0.614
MiNP	0.248 (-0.028, 0.525)	0.078	0.042 (0.004, 0.080)	0.032	-0.064 (-0.118, -0.010)	0.021	-0.062 (-0.115, -0.009)	0.023
MCHP	0.051 (-0.056, 0.158)	0.352	0.005 (-0.010, 0.020)	0.496	-0.004 (-0.025, 0.017)	0.721	-0.003 (-0.024, 0.017)	0.761
MEHP	0.010 (-0.110, 0.130)	0.872	0.018 (0.001, 0.034)	0.037	-0.013 (-0.037, 0.010)	0.272	-0.018 (-0.041, 0.005)	0.128
MECPP	0.373 (0.113, 0.632)	0.005	0.031 (-0.005, 0.067)	0.087	-0.049 (-0.100, 0.003)	0.062	-0.042 (-0.092, 0.008)	0.102
MEHHP	0.301 (0.024, 0.577)	0.033	0.043 (0.005, 0.081)	0.028	-0.078 (-0.132, -0.023)	0.005	-0.071 (-0.124, -0.018)	0.009
MEOHP	0.206 (-0.016, 0.428)	0.069	0.029 (-0.001, 0.060)	0.059	-0.062 (-0.105, -0.018)	0.005	-0.053 (-0.096, -0.010)	0.015
MCMHP	0.343 (0.107, 0.579)	0.004	0.032 (0.000, 0.065)	0.053	-0.058 (-0.104, -0.011)	0.015	-0.051 (-0.097, -0.005)	0.028

In the regression analysis, age, sex, BMI, current smoking status, and UIC were adjusted. Urinary concentrations of phthalate metabolites and UIC were corrected by urine Cr and ln-transformed to approximate a normal distribution.

**(a):** 

	TSH		FT_3_		FT_4_	
	Negative	Positive	Negative	Positive	Negative	Positive
MMP	-0.017 (-0.060, 0.027)	-0.180 (-0.409, 0.048)	0.019 (-0.020, 0.058)	-0.024 (-0.201, 0.154)	-0.028 (-0.179, 1.123)	0.476 (-0.409, 1.361)
MEP	-0.005 (-0.031, 0.021)	-0.043 (-0.195, 0.110)	-0.007 (-0.031, 0.016)	-0.018 (-0.134, 0.098)	-0.005 (-0.096, 0.086)	0.329 (-0.249, 0.906)
MiBP	0.006 (-0.058, 0.070)	-0.413 (-0.827, 0.004)	0.019 (-0.039, 0.078)	0.365 (0.055, 0.676)^∗^	-0.066 (-0.291, 0.159)	0.808 (-0.830, 2.447)
MnBP	0.017 (-0.040, 0.075)	-0.269 (-0.627, 0.090)	0.025 (-0.027, 0.078)	0.196 (-0.076, 0.469)	-0.216 (-0.417, -0.016)^∗^	0.708 (-0.680, 2.095)
MBzP	0.015 (-0.024, 0.054)	-0.024 (-0.299, 0.252)	0.005 (-0.030, 0.040)	0.056 (-0.153, 0.265)	0.018 (-0.118, 0.155)	-0.432 (-1.480, 0.615)
MCPP	0.012 (-0.011, 0.035)	-0.150 (-0.318, 0.017)	-0.013 (-0.034, 0.008)	0.010 (-0.122, 0.141)	-0.032 (-0.112, 0.049)	0.785 (0.165, 1.406)^∗^
MiNP	-0.030 (-0.085, 0.024)	-0.288 (-0.538, -0.038)^∗^	0.013 (-0.037, 0.062)	0.088 (-0.111, 0.286)	-0.132 (-0.323, 0.059)	0.675 (-0.315, 1.665)
MCHP	-0.004 (-0.024, 0.017)	-0.003 (-0.119, 0.112)	0.005 (-0.014, 0.024)	0.047 (-0.040, 0.134)	0.010 (-0.063, 0.083)	-0.223 (-0.660, 0.214)
MEHP	-0.011 (-0.035, 0.012)	-0.108 (-0.230, 0.013)	0.003 (-0.018, 0.024)	0.000 (-0.096, 0.095)	0.030 (-0.052, 0.112)	0.334 (-0.136, 0.804)
MECPP	-0.005 (-0.055, 0.045)	-0.515 (-0.808, -0.222)^∗∗^	0.024 (-0.022, 0.070)	0.261 (0.022, 0.500)^∗^	-0.180 (-0.356, -0.005)^∗^	1.016 (-0.213, 2.244)
MEHHP	-0.036 (-0.090, 0.017)	-0.453 (-0.744, 0.163)^∗∗^	0.021 (-0.028, 0.070)	0.193 (-0.043, 0.429)	-0.176 (-0.364, 0.012)	0.929 (-0.263, 2.120)
MEOHP	-0.028 (-0.070, 0.015)	-0.407 (-0.684, -0.130)^∗∗^	-0.009 (-0.048, 0.030)	0.168 (-0.056, 0.392)	-0.168 (-0.316, -0.019)^∗^	0.740 (-0.394, 1.875)
MCMHP	-0.025 (-0.071, 0.020)	-0.520 (-0.841, -0.199)^∗∗^	0.037 (-0.004, 0.079)	0.321 (0.068, 0.573)^∗^	-0.156 (-0.314, 0.003)	1.268 (-0.040, 2.575)

**(b):** 

	T_3_		T_4_		SPINA-G_D_	
	Negative	Positive	Negative	Positive	Negative	Positive
MMP	0.014 (-0.006, 0.034)	-0.020 (-0.088, 0.048)	-0.299 (-1.555, 0.958)	2.467 (-3.025, 7.960)	0.172 (-0.057, 0.402)	-0.476 (-1.328, 0.377)
MEP	-0.004 (-0.017, 0.008)	-0.030 (-0.074, 0.013)	-0.407 (-1.160, 0.345)	0.728 (-2.887, 4.343)	-0.014 (-0.152, 0.124)	-0.492 (-1.037, 0.054)
MiBP	0.003 (-0.027, 0.034)	0.082 (-0.041, 0.205)	-0.344 (-2.211, 1.523)	5.317 (-4.800, 15.434)	0.131 (-0.211, 0.473)	0.407 (-1.186, 1.999)
MnBP	0.011 (-0.016, 0.038)	0.050 (-0.055, 0.155)	-0.516 (-2.186, 1.154)	3.587 (-5.025, 12.199)	0.321 (0.017, 0.626)^∗^	0.159 (-1.193, 1.512)
MBzP	0.011 (-0.007, 0.029)	0.016 (-0.064, 0.096)	1.142 (0.014, 2.271)^∗^	-2.636 (-9.113, 3.842)	0.100 (-0.107, 0.307)	0.603 (-0.399, 1.605)
MCPP	-0.009 (-0.020, 0.002)	-0.025 (-0.075, 0.025)	-0.375 (-1.042, 0.291)	2.818 (-1.194, 6.831)	-0.052 (-0.174, 0.070)	-0.880 (-1.463, -0.296)^∗∗^
MiNP	0.018 (-0.007, 0.044)	-0.014 (-0.090, 0.063)	0.183 (-1.402, 1.768)	2.163 (-4.049, 8.375)	0.330 (0.041, 0.619)^∗^	-0.638 (-1.593, 0.318)
MCHP	0.004 (-0.005, 0.014)	0.024 (-0.009, 0.057)	0.557 (-0.047, 1.162)	-0.990 (-3.707, 1.728)	0.028 (-0.083, 0.139)	0.406 (-0.003, 0.815)
MEHP	0.005 (-0.006, 0.016)	-0.009 (-0.045, 0.027)	0.358 (-0.324, 1.039)	1.689 (-1.240, 4.619)	0.030 (-0.095, 0.155)	-0.313 (-0.767, 0.141)
MECPP	0.022 (-0.002, 0.046)	0.043 (-0.052, 0.138)	0.370 (-1.092, 1.831)	5.265 (-2.399, 12.929)	0.411 (0.145, 0.676)^∗∗^	-0.418 (-1.632, 0.796)
MEHHP	0.016 (-0.009, 0.041)	0.005 (-0.087, 0.098)	-0.054 (-1.619, 1.510)	3.783 (-3.695, 11.262)	0.350 (0.065, 0.635)^∗^	-0.699 (-1.860, 0.462)
MEOHP	0.007 (-0.013, 0.027)	0.011 (-0.077, 0.098)	-0.154 (-1.394, 1.086)	3.105 (-3.979, 10.189)	0.229 (0.002, 0.455)^∗^	-0.463 (-1.569, 0.642)
MCMHP	0.026 (0.004, 0.047)^∗^	0.014 (-0.089, 0.117)	-0.094 (-1.413, 1.224)	5.376 (-2.885, 13.637)	0.386 (0.147, 0.625)^∗^	-0.879 (-2.165, 0.407)

**(c):** 

	SPINA-G_T_		TSHI		TTSI	
	Negative	Positive	Negative	Positive	Negative	Positive
MMP	0.007 (-0.021, 0.036)	0.142 (-0.048, 0.332)	-0.020 (-0.065, 0.025)	-0.116 (-0.291, 0.058)	-0.019 (-0.062, 0.024)	-0.160 (-0.364, 0.044)
MEP	0.001 (-0.016, 0.018)	0.060 (-0.067, 0.186)	-0.005 (-0.032, 0.022)	0.001 (-0.115, 0.118)	-0.006 (-0.031, 0.020)	-0.031 (-0.168, 0.106)
MiBP	-0.008 (-0.050, 0.035)	0.360 (0.016, 0.704)^∗^	-0.003 (-0.070, 0.064)	-0.303 (-0.619, 0.013)	0.001 (-0.063, 0.065)	-0.390 (-0.760, -0.021)^∗^
MnBP	-0.016 (-0.054, 0.022)	0.227 (-0.070, 0.525)	-0.012 (-0.072, 0.048)	-0.174 (-0.447, 0.100)	0.004 (-0.054, 0.061)	-0.243 (-0.564, 0.077)
MBzP	0.003 (-0.023, 0.028)	-0.010 (-0.239, 0.220)	0.018 (-0.023, 0.058)	-0.082 (-0.290, 0.126)	0.017 (-0.022, 0.055)	-0.052 (-0.299, 0.194)
MCPP	-0.010 (-0.026, 0.005)	0.127 (-0.012, 0.266)	0.008 (-0.016, 0.032)	-0.044 (-0.175, 0.087)	0.010 (-0.013, 0.033)	-0.108 (-0.260, 0.044)
MiNP	0.022 (-0.014, 0.058)	0.216 (0.006, 0.426)^∗^	-0.048 (-0.105, 0.009)	-0.197 (-0.389, -0.006)^∗^	-0.039 (-0.093, 0.015)	-0.259 (-0.483, -0.036)^∗^
MCHP	0.006 (-0.008, 0.020)	-0.004 (-0.100, 0.092)	-0.002 (-0.024, 0.019)	-0.033 (-0.121, 0.054)	-0.003 (-0.024, 0.018)	-0.017 (-0.120, 0.087)
MEHP	0.010 (-0.005, 0.026)	0.079 (-0.023, 0.181)	-0.007 (-0.032, 0.017)	-0.064 (-0.157, 0.030)	-0.010 (-0.033, 0.014)	-0.091 (-0.201, 0.018)
MECPP	0.010 (-0.023, 0.043)	0.402 (0.154, 0.650)^∗∗^	-0.029 (-0.082, 0.023)	-0.378 (-0.603, -0.154)	-0.017 (-0.067, 0.033)	-0.473 (-0.734, -0.212)^∗∗^
MEHHP	0.024 (-0.011, 0.060)	0.340 (0.094, 0.586)^∗∗^	-0.060 (-0.116, -0.004)^∗^	-0.329 (-0.551, -0.106)^∗∗^	-0.048 (-0.101, 0.005)	-0.414 (-0.673, -0.156)^∗∗^
MEOHP	0.017 (-0.011, 0.045)	0.303 (0.068, 0.537)^∗^	-0.050 (-0.094, -0.006)^∗^	-0.308 (-0.518, -0.097)^∗∗^	-0.038 (-0.080, 0.004)	-0.378 (-0.624, -0.132)^∗∗^
MCMHP	0.017 (-0.013, 0.047)	0.417 (0.148, 0.685)^∗∗^	-0.046 (-0.093, 0.001)	-0.349 (-0.599, -0.100)^∗∗^	-0.035 (-0.080, 0.010)	-0.466 (-0.753, -0.180)^∗∗^

In the multiple regression analysis, age, sex, BMI, current smoking status, and UIC were adjusted. Urinary concentrations of phthalate metabolites and UIC were corrected by urine Cr and ln-transformed to approximate a normal distribution. ^∗^*P* < 0.05, ^∗∗^*P* < 0.01, and ^∗∗∗^*P* < 0.001. “Negative”: TPOAb (-) and TgAb (-); “positive”: TPOAb (+) and/or TgAb (+).

**(a):** 

	TSH		FT_3_		FT_4_		T_3_		T_4_	
	*B* (95% CI)	*P*	*B* (95% CI)	*P*	*B* (95% CI)	*P*	*B* (95% CI)	*P*	*B* (95% CI)	*P*
Factor 1	-0.056 (-0.107, -0.004)	0.034	0.010 (-0.036, 0.056)	0.670	-0.051 (-0.233, 0.132)	0.586	0.014 (-0.009, 0.037)	0.225	0.575 (-0.885, 2.036)	0.440
Factor 2	0.189 (-0.044, 0.422)	0.111	-0.033 (-0.240, 0.174)	0.753	-0.190 (-1.019, 0.638)	0.652	-0.058 (-0.161, 0.046)	0.276	-3.196 (-9.820, 3.428)	0.344
Factor 3	-0.026 (-0.073, 0.020)	0.269	0.019 (-0.023, 0.060)	0.371	0.045 (-0.121, 0.211)	0.592	0.011 (-0.009, 0.032)	0.284	0.507 (-0.818, 1.833)	0.453
Factor 4	0.001 (-0.002, 0.010)	0.202	-0.004 (-0.046, 0.038)	0.863	-0.056 (-0.224, 0.122)	0.512	0.004 (-0.017, 0.025)	0.710	-0.506 (-1.848, 0.836)	0.459

**(b):** 

	SPINA-G_D_		SPINA-G_T_		TSHI		TTSI	
	*B* (95% CI)	*P*	*B* (95% CI)	*P*	*B* (95% CI)	*P*	*B* (95% CI)	*P*
Factor 1	0.209 (-0.051, 0.469)	0.115	0.043 (0.007, 0.079)	0.018	-0.062 (-0.114, -0.011)	0.017	-0.060 (-0.110, -0.010)	0.019
Factor 2	-0.424 (-1.603, 0.756)	0.481	-0.144 (-0.306, 0.018)	0.082	0.163 (-0.068, 0.395)	0.166	0.177 (-0.050, 0.404)	0.126
Factor 3	0.083 (-0.153, 0.319)	0.489	0.022 (-0.010, 0.055)	0.180	-0.020 (-0.066, 0.026)	0.393	-0.024 (-0.069, 0.022)	0.309
Factor 4	0.122 (-0.117, 0.362)	0.315	-0.007 (-0.040, 0.026)	0.684	-0.006 (-0.053, 0.041)	0.795	-0.002 (-0.048, 0.044)	0.920

Adjusted for age, sex, BMI, smoking status, and urine iodine to creatinine ratio and Factors 1, 2, 3, and 4. Factor 1 was highly loaded with MECPP, MEHHP, MEOHP, MCMHP, and MiNP. Factor 2 was highly loaded with MiBP, MnBP, and MEHP. Factor 3 was highly loaded with MCPP and MCHP, and Factor 4 was highly loaded with MBzP.

**(a):** 

	TSH		FT_3_		FT_4_	
	Metformin	Nonmetformin	Metformin	Nonmetformin	Metformin	Nonmetformin
MMP	-0.017 (-0.077, 0.044)	-0.045 (-0.110, 0.019)	0.021 (-0.030, 0.072)	0.019 (-0.040, 0.077)	-0.012 (0.216, 0.192)	0.083 (-0.149, 0.314)
MEP	-0.018 (-0.062, 0.025)	0.003 (-0.032, 0.038)	-0.011 (-0.047, 0.026)	-0.008 (-0.040, 0.023)	-0.181 (-0.325, -0.036)^∗^	0.133 (0.007, 0.258)^∗^
MiBP	-0.019 (-0.123, 0.086)	-0.005 (-0.095, 0.084)	0.039 (-0.049, 0.126)	0.032 (-0.049, 0.113)	-0.142 (-0.492, 0.209)	0.101 (-0.220, 0.423)
MnBP	-0.049 (-0.143, 0.046)	0.032 (-0.046, 0.111)	0.010 (-0.070, 0.089)	0.047 (-0.024, 0.118)	-0.145 (-0.463, 0.173)	-0.106 (-0.389, 0.176)
MBzP	0.010 (-0.054, 0.074)	0.016 (-0.038, 0.069)	0.052 (-0.001, 0.105)	-0.015 (-0.064, 0.034)	0.135 (-0.080, 0.349)	-0.104 (-0.298, 0.090)
MCPP	-0.001 (-0.037, 0.034)	0.013 (-0.019, 0.046)	-0.008 (-0.038, 0.021)	-0.015 (-0.045, 0.015)	0.079 (-0.041, 0.198)	-0.073 (-0.190, 0.044)
MiNP	-0.067 (-0.153, 0.019)	-0.044 (-0.119, 0.031)	0.049 (-0.023, 0.121)	0.002 (-0.066, 0.070)	-0.329 (-0.615, -0.043)^∗^	0.105 (-0.166, 0.376)
MCHP	-0.019 (-0.050, 0.012)	0.009 (-0.021, 0.039)	0.018 (-0.008, 0.045)	-0.003 (-0.030, 0.024)	0.009 (-0.097, 0.115)	-0.028 (-0.135, 0.079)
MEHP	0.009 (-0.027, 0.044)	-0.042 (-0.074, -0.009)^∗^	0.016 (-0.014, 0.046)	-0.002 (-0.032, 0.027)	-0.040 (-0.160, 0.080)	0.127 (0.011, 0.244)^∗^
MECPP	-0.045 (-0.131, 0.042)	-0.028 (-0.097, 0.040)	0.106 (0.034, 0.177)^∗∗^	0.004 (-0.058, 0.066)	-0.383 (-0.671, -0.096)^∗∗^	0.017 (-0.229, 0.263)
MEHHP	-0.072 (-0.156, 0.011)	-0.055 (-0.131, 0.021)	0.061 (-0.009, 0.132)	0.017 (-0.052, 0.086)	-0.365 (-0.644, -0.086)^∗^	0.069 (-0.205, 0.344)
MEOHP	-0.059 (-0.135, 0.016)	-0.037 (-0.093, 0.020)	0.053 (-0.010, 0.116)	-0.028 (-0.079, 0.024)	-0.271 (-0.523, -0.018)^∗^	-0.082 (-0.286, 0.123)
MCMHP	-0.050 (-0.121, 0.021)	-0.039 (-0.104, 0.027)	0.083 (0.024, 0.142)^∗∗^	0.021 (-0.039, 0.081)	-0.372 (-0.607, -0.137)^∗∗^	0.081 (-0.156, 0.319)

**(b):** 

	T_3_		T_4_		SPINA-G_D_	
	Metformin	Nonmetformin	Metformin	Nonmetformin	Metformin	Nonmetformin
MMP	0.025 (-0.001, 0.052)	0.001 (-0.027, 0.029)	0.467 (-1.218, 2.152)	-0.354 (-2.141, 1.433)	0.269 (-0.054, 0.591)	-0.029 (-0.336, 0.277)
MEP	-0.008 (-0.027, 0.011)	-0.006 (-0.022, 0.009)	-1.327 (-2.521, 0.132)^∗^	0.260 (-0.710, 1.231)	0.132 (-0.099, 0.363)	-0.158 (-0.324, 0.008)
MiBP	0.007 (-0.038, 0.053)	0.006 (-0.033, 0.046)	-1.051 (-3.944, 1.843)	0.865 (-1.616, 3.346)	0.193 (-0.363, 0.750)	0.052 (-0.374, 0.477)
MnBP	-0.004 (-0.046, 0.037)	0.023 (-0.012, 0.057)	-1.177 (-3.800, 1.446)	0.555 (-1.625, 2.735)	0.078 (-0.427, 0.583)	0.361 (-0.011, 0.733)
MBzP	0.034 (0.006, 0.062)^∗^	0.001 (-0.023, 0.024)	2.598 (0.852, 4.343)^∗∗^	-0.044 (-1.544, 1.457)	0.255 (-0.115, 0.565)	0.110 (-0.147, 0.367)
MCPP	-0.005 (-0.020, 0.011)	-0.015 (-0.029, 0.000)^∗^	0.372 (-0.617, 1.360)	-0.910 (-1.811, -0.010)^∗^	-0.118 (-0.308, 0.072)	-0.052 (-0.207, 0.103)
MiNP	0.024 (-0.013, 0.061)	0.008 (-0.025, 0.041)	-1.053 (-3.435, 1.328)	1.174 (-0.914, 3.262)	0.557 (0.104, 1.011)^∗^	0.020 (-0.338, 0.379)
MCHP	0.008 (-0.005, 0.022)	0.004 (-0.009, 0.017)	0.430 (-0.442, 1.302)	0.501 (-0.324, 1.326)	0.036 (-0.132, 0.204)	0.070 (-0.072, 0.211)
MEHP	0.009 (-0.007, 0.024)	0.004 (-0.010, 0.018)	0.132 (-0.862, 1.125)	0.871 (-0.031, 1.774)	0.131 (-0.059, 0.322)	-0.063 (-0.218, 0.093)
MECPP	0.045 (0.008, 0.083)^∗^	0.012 (-0.018, 0.042)	-0.876 (-3.277, 1.526)	1.357 (-0.534, 3.248)	0.791 (0.339, 1.243)^∗∗^	0.148 (-0.177, 0.473)
MEHHP	0.022 (-0.014, 0.059)	0.012 (-0.021, 0.046)	-1.339 (-3.667, 0.989)	1.154 (-0.959, 3.268)	0.568 (0.125, 1.012)^∗^	0.097 (-0.265, 0.460)
MEOHP	0.021 (-0.012, 0.054)	0.000 (-0.025, 0.025)	-1.047 (-3.144, 1.051)	0.296 (-1.285, 1.877)	0.465 (0.065, 0.865)^∗^	0.073 (-0.198, 0.344)
MCMHP	0.035 (0.004, 0.066)^∗^	0.018 (-0.011, 0.047)	-1.344 (-3.314, 0.626)	1.214 (-0.615, 3.042)	0.599 (0.226, 0.972)^∗∗^	0.154 (-0.160, 0.467)

**(c):** 

	SPINA-G_T_		TSHI		TTSI	
	Metformin	Nonmetformin	Metformin	Nonmetformin	Metformin	Nonmetformin
MMP	0.014 (-0.025, 0.053)	0.025 (-0.022, 0.072)	-0.018 (-0.083, 0.046)	-0.034 (-0.094, 0.026)	-0.018 (-0.079, 0.043)	-0.042 (-0.103, 0.019)
MEP	0.003 (-0.025, 0.031)	0.003 (-0.022, 0.029)	-0.043 (-0.088, 0.003)	0.021 (-0.011, 0.054)	-0.030 (-0.074, 0.013)	0.010 (-0.023, 0.043)
MiBP	-0.001 (-0.068, 0.066)	0.014 (-0.051, 0.080)	-0.038 (-0.148, 0.073)	0.008 (-0.075, 0.092)	-0.027 (-0.132, 0.077)	-0.002 (-0.087, 0.083)
MnBP	0.010 (-0.051, 0.070)	-0.010 (-0.067, 0.048)	-0.068 (-0.168, 0.032)	0.018 (-0.055, 0.091)	-0.057 (-0.152, 0.038)	0.024 (-0.051, 0.098)
MBzP	0.018 (-0.023, 0.059)	-0.008 (-0.048, 0.031)	0.029 (-0.039, 0.096)	0.002 (-0.049, 0.052)	0.018 (-0.046, 0.083)	0.010 (-0.042, 0.061)
MCPP	0.005 (-0.017, 0.028)	-0.016 (-0.040, 0.008)	0.009 (-0.028, 0.047)	0.003 (-0.027, 0.034)	0.003 (-0.032, 0.039)	0.008 (-0.023, 0.039)
MiNP	0.029 (-0.027, 0.084)	0.044 (-0.011, 0.099)	-0.111 (-0.201, -0.021)^∗^	-0.030 (-0.100, 0.041)	-0.088 (-0.173, -0.002)^∗^	-0.039 (-0.111, 0.032)
MCHP	0.014 (-0.006, 0.034)	-0.001 (-0.023, 0.021)	-0.018 (-0.051, 0.015)	0.005 (-0.023, 0.033)	-0.018 (-0.049, 0.014)	0.007 (-0.021, 0.036)
MEHP	-0.004 (-0.027, 0.019)	0.032 (0.009, 0.056)^∗∗^	0.003 (-0.035, 0.041)	-0.025 (-0.055, 0.006)	0.006 (-0.030, 0.042)	-0.034 (-0.065, -0.003)^∗^
MECPP	0.019 (-0.037, 0.074)	0.035 (-0.015, 0.085)	-0.096 (-0.188, -0.005)^∗^	-0.026 (-0.090, 0.038)	-0.069 (-0.155, 0.018)	-0.029 (-0.094, 0.036)
MEHHP	0.033 (-0.021, 0.086)	0.047 (-0.009, 0.103)	-0.121 (-0.209, -0.034)^∗∗^	-0.046 (-0.117, 0.025)	-0.095 (-0.179, -0.011)^∗^	-0.053 (-0.125, 0.019)
MEOHP	0.022 (-0.026, 0.071)	0.029 (-0.013, 0.070)	-0.096 (-0.175, -0.016)^∗^	-0.048 (-0.101, 0.005)	-0.076 (-0.152, -0.001)^∗^	-0.042 (-0.096, 0.012)
MCMHP	0.019 (-0.026, 0.065)	0.038 (-0.010, 0.086)	-0.100 (-0.175, -0.026)^∗∗^	-0.028 (-0.089, 0.034)	-0.072 (-0.143, -0.001)^∗^	-0.035 (-0.098, 0.027)

In the multiple regression analysis, age, sex, BMI, current smoking status, and UIC were adjusted. Urinary concentrations of phthalate metabolites and UIC were corrected by urine Cr and ln-transformed to approximate a normal distribution. ^∗^*P* < 0.05, ^∗∗^*P* < 0.01, and ^∗∗∗^*P* < 0.001.

## Data Availability

The data used to support the findings of this study are included within the article.
